# Hopping in hypogravity—A rationale for a plyometric exercise countermeasure in planetary exploration missions

**DOI:** 10.1371/journal.pone.0211263

**Published:** 2019-02-13

**Authors:** Tobias Weber, David A. Green, Julia Attias, Wolfram Sies, Alexandre Frechette, Bjoern Braunstein, Jörn Rittweger

**Affiliations:** 1 European Space Agency, European Astronaut Centre, Space Medicine Team (HRE-OM), Cologne, Germany; 2 KBRwyle GmbH, Cologne, Germany; 3 King’s College London, Centre of Applied Physiological Sciences (CHAPS), London, United Kingdom; 4 Institute of Aerospace Medicine, German Aerospace Center (DLR), Cologne, Germany; 5 Institute of Biomechanics and Orthopaedics, German Sport University, Cologne, Germany; 6 Centre for Health and Integrative Physiology in Space (CHIPS), Cologne, Germany; 7 Department of Pediatrics and Adolescent Medicine, University of Cologne, Cologne, Germany; Fondazione Santa Lucia Istituto di Ricovero e Cura a Carattere Scientifico, ITALY

## Abstract

Moon and Mars are considered to be future targets for human space explorations. The gravity level on the Moon and Mars amount to 16% and 38%, respectively, of Earth’s gravity. Mechanical loading during the anticipated habitual activities in these hypogravity environments will most likely not be sufficient to maintain physiological integrity of astronauts unless additional exercise countermeasures are performed. Current microgravity exercise countermeasures appear to attenuate but not prevent ‘space deconditioning’. However, plyometric exercises (hopping and whole body vibration) have shown promise in recent analogue bed rest studies and may be options for space exploration missions where resources will be limited compared to the ISS. This paper therefore tests the hypothesis that plyometric hop exercise in hypogravity can generate sufficient mechanical stimuli to prevent musculoskeletal deconditioning. It has been suggested that hypogravity-induced reductions in peak ground reaction force (peak vertical GRF) can be offset by increases in hopping height. Therefore, this study investigated the effects of simulated hypogravity (0.16G, 0.27G, 0.38G, and 0.7G) upon sub-maximal plyometric hopping on the Verticalised Treadmill Facility, simulating different hypogravity levels. Results show that peak vertical GRF are negatively related to simulated gravity level, but positively to hopping height. Contact times decreased with increasing gravity level but were not influenced through hopping height. In contrast, flight time increased with decreasing gravity levels and increasing hopping height (*P* < 0.001). The present data suggest that the anticipated hypogravity-related reductions of musculoskeletal forces during normal walking can be compensated by performing hops and therefore support the idea of plyometric hopping as a robust and resourceful exercise countermeasure in hypogravity. As maximal hop height was constrained on the VTF further research is needed to determine whether similar relationships are evident during maximal hops and other forms of jumping.

## Introduction

Five decades after Neil Armstrong set foot on the Moon, International Space Agencies are once again looking at deep space exploration. The European Space Agency (ESA) recently announced that it intends to send humans back to the lunar surface. Thus, ESA has recently embarked upon scoping activities in preparation of potential establishment of a habitat at one of the lunar poles (the so-called “Moon Village”) to facilitate deep space research and technology demonstrations [1]. Once a habitat on the lunar surface is established, typical mission durations are anticipated to be around 42 days [2], far exceeding the longest Apollo era surface time of 75 hours (Apollo 17).

Whilst, since the Apollo programme significant knowledge regarding the operational and physiological effects of living and working in microgravity (μG) has been accumulated [3, 4], the effect of longer periods in lunar gravity (16% of Earth’s gravity) remains unknown [5]. Some authors have purported that lunar gravity will be insufficient to maintain musculoskeletal integrity, due to a concomitant reduction in the mechanical stimuli associated with movement that have been suggested to be key stimuli for muscle and bone regulation [5, 6]. However, whether lunar gravity is sufficient to prevent against physical deconditioning similar to that observed in μG remains to be determined.

In fact, muscle atrophy and bone loss induced by bed rest (the current ‘gold standard’ ground-based analogue to simulate the physiological effects of μG) has been shown to be reduced when forceful muscle contractions are performed daily [7, 8]. However, this is not true when muscle contractions are performed only every other day [9], or sub-maximally [10]. Therefore, controlled production of forceful, approximating maximal contractions within lunar gravity are likely to be advantageous in maintaining musculoskeletal integrity.

Current exercise countermeasures on-board the International Space Station (ISS) comprise of approximately 90 minutes of actual exercise time per day. The prescribed (concurrent) exercise program involves both resistive and aerobic exercise using a number of bulky devices such as the T2 treadmill, where for the majority of astronauts and exercise sessions loads between 0.7G and 1G are provided via bungee ropes [11]. Such measures have been shown to ameliorate physiological space deconditioning such as loss of bone mineral density, aerobic capacity and muscle strength in most, albeit not all crewmembers during 6 month ISS missions [12]. However, hardware requirements in lunar missions will most likely be significantly more restrictive in terms of upload mass, size and robustness [13, 14]. Therefore, any exercise employed within a lunar habitat must not only be safe, efficient and effective but should require no, or minimal hardware that is lightweight, compact, easy to set up, store and maintain for long periods.

Plyometric exercise—defined as movement involving repetitive and short duration-high force loading has been proposed as an effective way to load the musculoskeletal system and has been shown to improve muscle function (primarily muscle power) and bone strength even in healthy individuals [15, 16]. Peak vertical ground reaction forces (peak vertical GRF) are closely related to resultant bone deformation [17, 18] and thus strain with bone strain magnitude suggested to govern bone’s mechano-adaptation (Kriechbaumer et al, under revision). Thus, lunar countermeasures targeting (at least in part) bone maintenance should seek to involve bone strains of similar magnitude to those experienced on Earth.

Finite element 3D modelling of bone deformation during various exercises suggests that hopping (a form of plyometric exercise) generates very high tibial deformation compared to other exercise forms (Kriechbaumer et al., under review). These findings potentially explain the fact that recently repeated hopping performed within a pressure cylinder-based sledge jump system entirely ameliorated musculoskeletal de-conditioning induced during 60-day head down bed-rest [8]. Moreover, whole body vibration, which similarly leads to stretch-shortening cycles as hopping [19] has additional benefit against musculoskeletal de-conditioning when superimposed on resistive exercise [20]. However, peak vertical GRF associated with such hops were not quantified in previous biomechanical hypogravity studies [5].

Following Newton’s logic, jump height is a function of the vertical impulse, which is the integral of vertical ground reaction force with time (the interplay of mechanical key features determining hopping height is presented in the supporting information S1 Fig). There are only two studies available that have investigated the effects of hopping or jumping in hypogravity. In a hypogravity parabolic flight campaign participants rapidly adapted to changing gravity conditions by adjusting neuro-motor control of lower leg muscles [21] with peak reaction forces increasing with gravity level, and contact times decreasing. However, this study failed to account for changes in jump (flight) height. In fact, there was limited headspace (approx. 0.7m) and jump height was not standardized or measured. Jump height is critical as Cavagna et al. [22] investigated the mechanical characteristics of “jumping on the Moon” using an upright suspension system from which they predicted that the maximal counter movement HoF in lunar gravity conditions could be 4.1m, with a flight time of approximately 5 seconds.

However, it is unknown whether humans will achieve their maximal jump height through changes in push-time or push-force when in hypogravity. When performing plyometric hops where leg extension is limited similar to that during sledge jumping [8] we hypothesized that push-force would be the key determinant, whereas vertical impulse magnitude is more related to vertical displacement (height of flight—HoF) than contact times but that they are all scaled to hopping height and gravity.

Therefore, the aim of this study was to determine the biomechanical features, and resultant peak vertical GRF (as a predictor of bone strain) associated with sub-maximal bipedal hopping in simulated partial gravities that correspond to Lunar (0.16G), Martian (0.38G), 0.27G (equi-distant between Lunar and Martian) and 0.7G (the average harness load that is used during treadmill running on the ISS [11]). We hypothesized that: a) peak vertical GRF decreases with gravity, b) peak vertical GRF increases with hopping height, and c) that hypogravity-related reductions in peak vertical GRF can be compensated by increasing hopping height.

## Methods

### Participants

Eight healthy male participants (29.4±5.2years; 78.6 ± 6.8kg; 176.4 ± 6.7cm), gave written informed consent to participate in this study that received approval from the Nordrhein Medical Association in Düsseldorf (Germany). All experiments were conducted to the standards set out by the latest revision of the Declaration of Helsinki (2013) in a single session in the Physiology Laboratory of the Institute of Aerospace Medicine at the German Aerospace Center (DLR; Cologne, Germany). Participants were required to hop on the Verticalised Treadmill Facility (VTF) for 3x30s trials at each of four simulated hypogravity levels (0.7G, 0.38G, 0.27G, and 0.16G) in a randomized order.

Prior to the experimental session, all participants provided a resting 12-lead Electrocardiogram (ECG) that were read and evaluated by a qualified clinician, before being cleared to participate. All participants also visited the on-site physician for a medical examination on the day of the study, which consisted of a medical history evaluation, resting blood pressure-, heart and respiration- (rate and sounds) and standard anthropometric tests. Furthermore, they were all recreationally active and denied taking any medication and did not report any current or significant history of neurological, cardiorespiratory or musculoskeletal disorders.

### The vertical treadmill facility (VTF)

**Fig 1:**
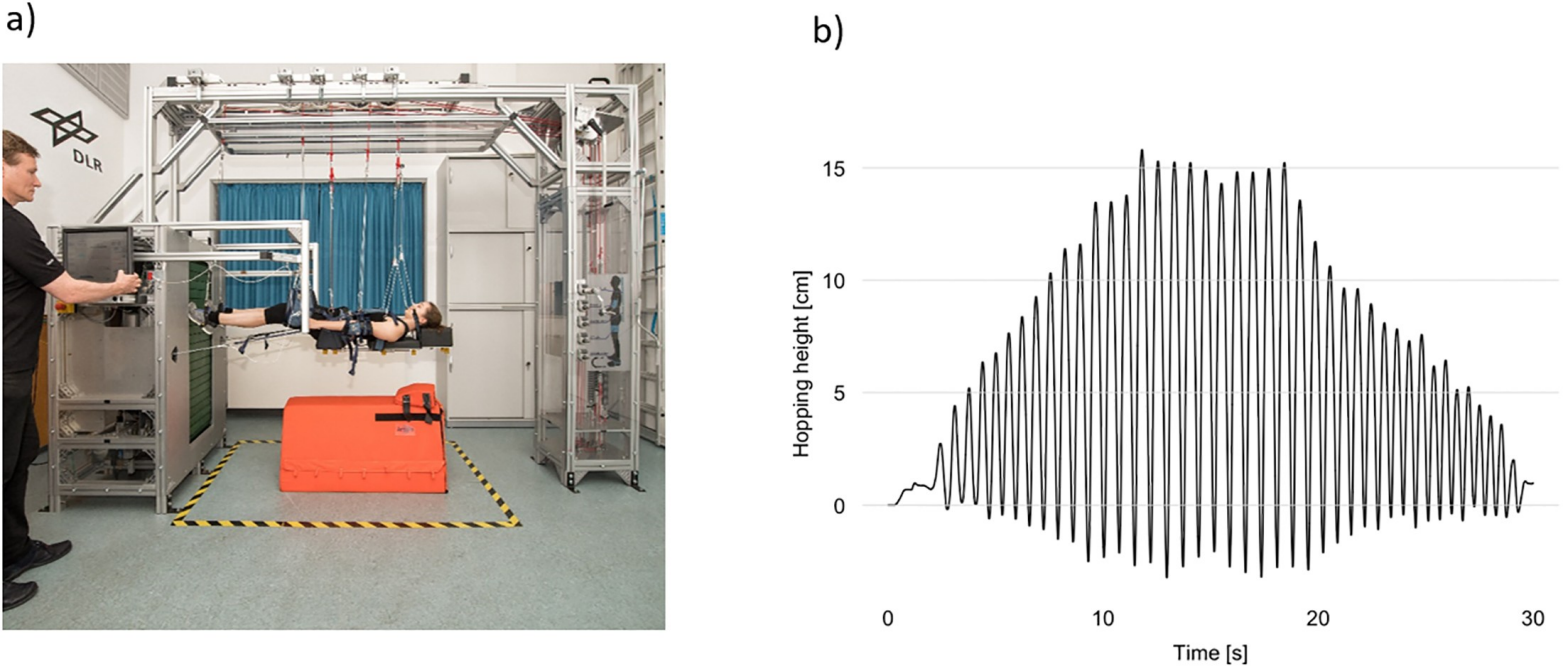
The vertical treadmill facility (VTF). Panel a shows the setup of the VTF. The elongation of pistons of one 30s hopping trial at 0.38g is shown in Panel b. The elongation of the VTF pistons was used to calculate height of flight (hopping height/jump height).

The VTF (Arsalis, Glabais, Belgium, Fig 1) consists of:

A verticalized treadmill: a customized, commercially available treadmill (Woodway, Waukesha, WI, USA) mounted vertically into a chassis in a manner similar to that used in the T2 on board the ISS.
A suspension system: provides suspension of the subject via a harness, strings and slings attached to an adjustable spring-loading system to offset terrestrial gravity.
The subject loading system (SLS) generates a pull-down-force equivalent to the average person’s body mass (Range: 180–990 N) based on the product of piston pressure and piston cross-section [23]. As the piston’s cross section is small in relation to its’ volume, the force variation during a normal running cycle with displacement ≤ 10cm approximates 5%.

### Study protocol

Prior to being suspended on the VTF, participants’ mass was measured (scale) to determine the required SLS load for that individual at a given hypogravity (5N VTF control increments). Participants were asked to bi-laterally hop for 3x30s trials on the VTF at each of the four simulated hypogravity levels in a randomized order whilst instructed to keep their knees stiff and contact times as short as possible (consistent with Kramer et al. [8]).

Before starting the plyometric hops, each participants’ head vertex was projected onto a back board with a laser, and the location marked with an additional piece of tape placed 20cm ‘above’ this point to mark their target maximal hop height. During the protocol for each 30s trial, participants were instructed over 10s to progressively increase from a low amplitude hop to their target hop height, to then hop at that height for 10s, before progressively reducing hop height for the final 10s (see Fig 1 and video in online supporting information S1 Video). Subjects were asked to hop exclusively using the balls of their feet, and not their heels which was easily achievable as only hop heights up to 20 cm were required. To aid the participants, they were provided with real time visual feedback of their hop height via a TV-screen linked to a camera (GoPro, Inc., San Mateo, CA, USA) focused on the target maximal hop height indicator and an audio file (supporting information S1 Video) was played to guide hopping height.

Before data was recorded participants were fully familiarized with the task and their performance was assessed by an experienced exercise specialist.

### Data acquisition

Ground reaction forces were measured using four in-built Mini-3D load cells (Arsalis, Glabais, Belgium) mounted to the chassis under the treadmill. Each individual load cell signal (Fx, Fy, Fz) was used to derive a composite Fz signal (peak vertical GRF) sampled at 250Hz and stored on the VTF-internal computer.

### Data processing

All data files were exported from the VTF computer for offline analysis. From the composite Fz signal: Absolute peak hopping reaction forces (Peal vertical GRF), time of the impulse (contact time—Tc) and flight time (Tf) were calculated. SLS piston displacement was used to calculate height of flight (HoF; defined as the difference between greatest and smallest piston excursion for each hopping cycle).

Peak vertical GRF signals were automatically segmented at 50% of the standing force, to differentiate flight and contact phases with custom-written R-scripts (www.r-project.org). All signals were visually inspected before peak vertical GRF, Tc, Tf and HoF were computed for each hopping cycle from the segmented data.

The modulation of pull-down force during hopping cycles for different pre-set G-levels is expressed as means over subjects and their standard deviation. Modulation of pull-down force was computed in % as 100·(maximum-minimum)/mean of the pull-down force for each hopping cycle.

### Statistics

Linear mixed effect models were constructed with participant as a random factor, gravity level and HoF as independent variables, and peak vertical GRF, Tc and Tf as dependent variables. These models yielded satisfactory residual and quantile-quantile plots, so no data transformation was necessary. G-levels and pull-down forces are presented as means and standard deviations (SD).

## Results

The data of one participant (A) was discarded due to inappropriately set VTF pull-back forces (see Table 1), which had been noticed only after the study had been closed.

**Table 1:** Pre-set g-levels vs. actual g-levels, expressed as subject individual means (SD).

Preset G	0.16 G	0.27G	0.38G	0.7G
**Actual G—subject A**	*0*.*29 (0)*	*0*.*4 (0)*	*0*.*5 (0)*	*0*.*78 (0)*
**Actual G—subject B**	0.15 (0)	0.27 (0)	0.37 (0)	0.66 (0)
**Actual G—subject C**	0.16 (0)	0.23 (0)	0.37 (0.01)	0.68 (0)
**Actual G—subject D**	0.18 (0)	0.29 (0)	0.38 (0)	0.69 (0.01)
**Actual G—subject E**	0.15 (0.01)	0.27 (0)	0.36 (0)	0.67 (0)
**Actual G—subject F**	0.16 (0)	0.26 (0)	0.34 (0.01)	0.64 (0.01)
**Actual G—subject G**	0.16 (0)	0.25 (0)	0.38 (0)	0.63 (0)
**Actual G—subject H**	0.19 (0)	0.3 (0)	0.41 (0)	0.71 (0)

**Fig 2:**
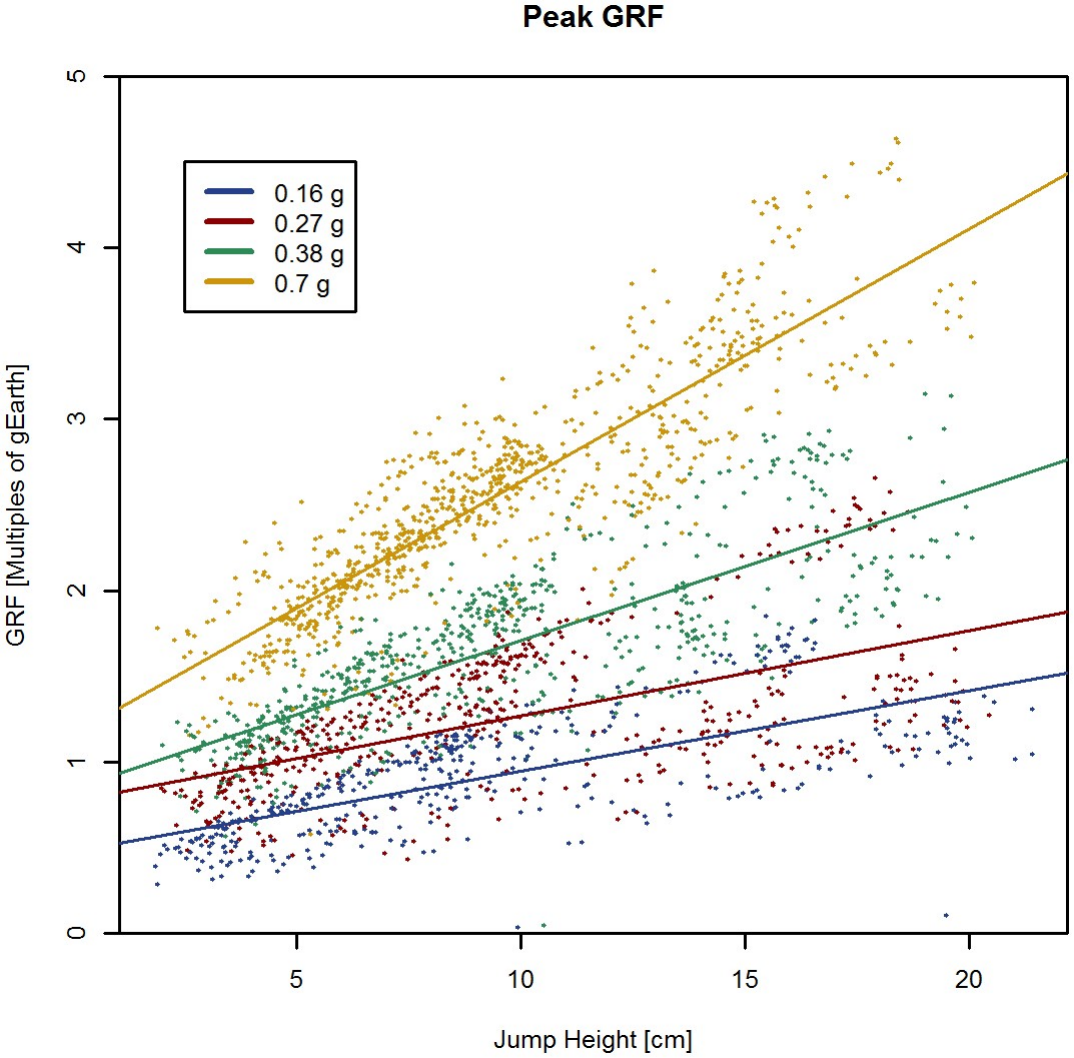
Hopping peak reaction forces (peak vertical GRF). Composite peak vertical GRF signals of all hopping trials are depicted. Ground Reaction Forces (GRF) are expressed as multiples of Earth’s gravity (1g). Peak vertical GRF increased significantly with increasing gravity levels and increasing jump heights (height of flight; *P* < 0.001).

Peak vertical GRF negatively related to simulated hypogravity level (*P* < 0.001), but positively related to hopping height (*P* < 0.001) with a significant interaction effect (*P* < 0.001; Fig 2 shows the composite signal of all participants and individual plots are presented in supporting information S2 Fig).

**Fig 3:**
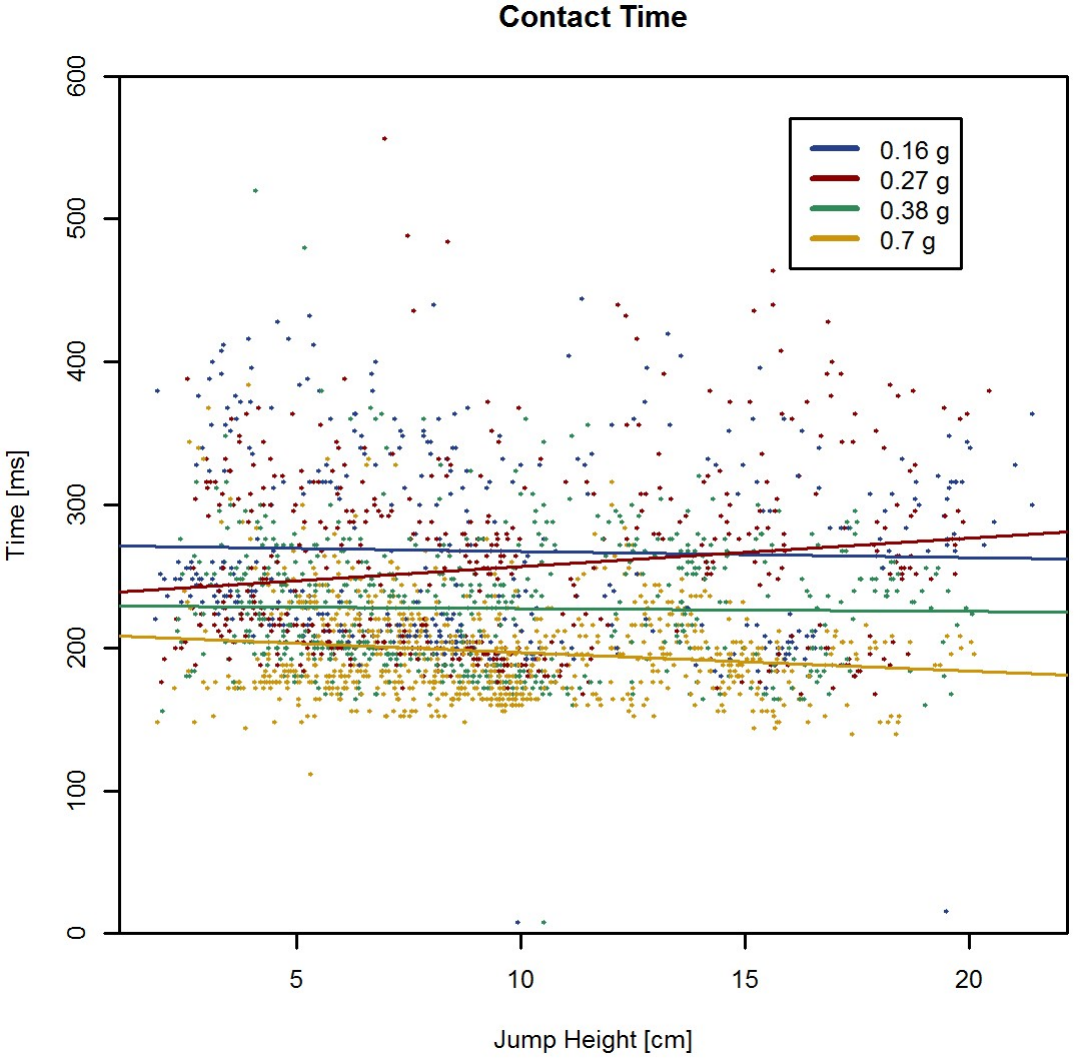
Contact time (Tc). Composite Tc signals of all hopping trials are depicted. Contact time decreased significantly with increasing gravity levels (*P* < 0.001) while jump heights (height of flight) had no effect on contact time.

Contact times decreased significantly with increasing simulated hypogravity levels (*P* < 0.001) but were not affected by HoF (Fig 3 and supporting information S3 Fig).

**Fig 4:**
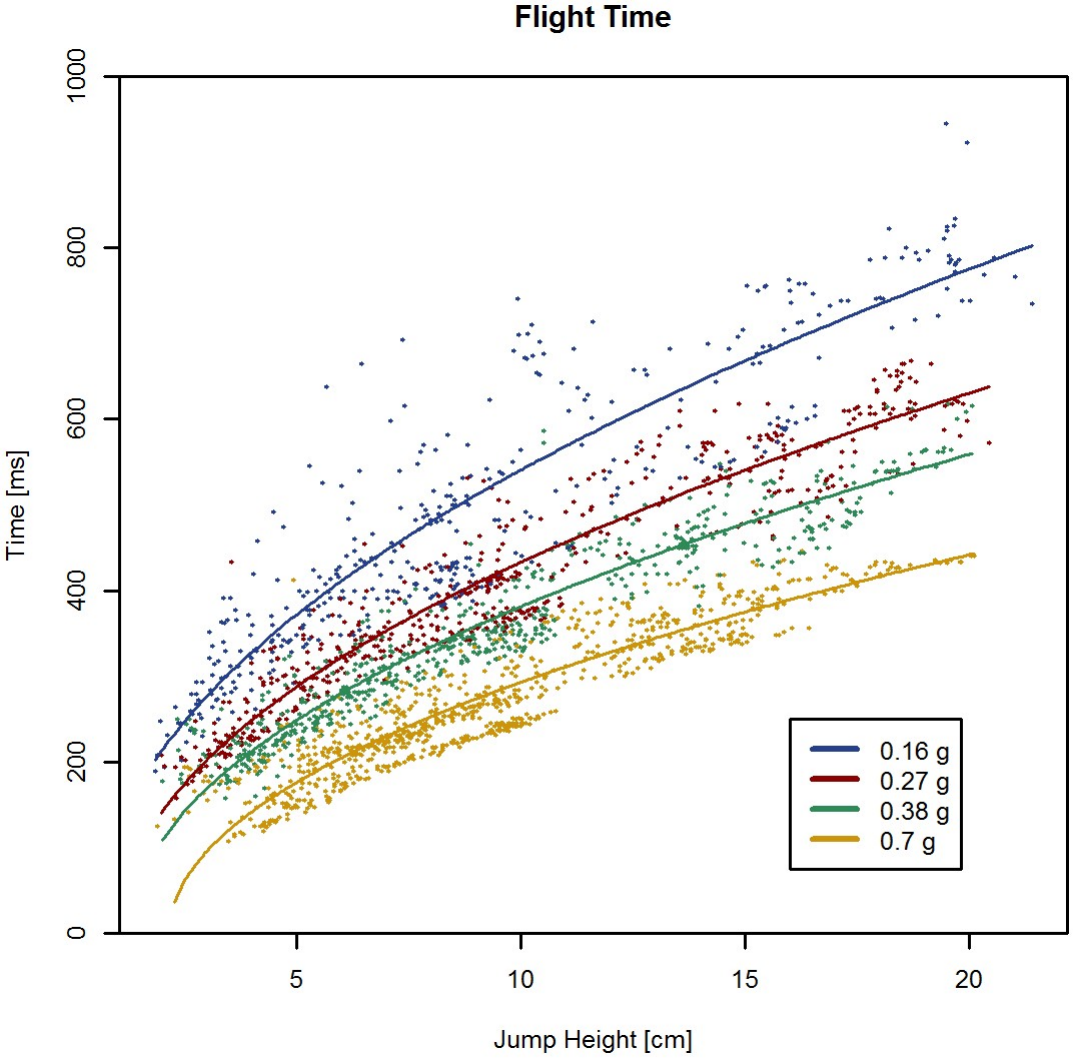
Flight time (Tf). Composite Tf signals of all hopping trials are depicted. A square-root function has been fitted to the data. Flight time increased significantly with decreasing gravity levels and increasing jump heights (height of flight; *P* < 0.001).

Flight time was affected by HoF, by simulated hypogravity level, and by their interaction (all *P* < 0.001). Flight time increased with decreasing simulated hypogravity levels and increasing hopping height (*P* < 0.001; Fig 4 and supporting information S4 Fig).

The modulation of pull-down force varied from 81.6% (SD = 20.2%) at 0.16G, to 64.5% (SD = 12.1%) at 0.27G, to 56.3% (SD = 10.4%) at 0.38% and to 44.9% (SD = 7.7%) at 0.7G.

## Discussion

This study set out with the idea that hypogravity-related reductions in GRF can be compensated by increasing hopping height. The main finding, namely peak reaction forces during hopping increase with increasing gravity levels and increasing hopping height confirm this concept. The present data also show that contact times decrease with increasing hypogravity levels but are not influenced by hopping height, while flight time increases with decreasing hypogravity levels and increasing hopping height.

Performing exercise under hypogravity conditions, *e*.*g*. in a lunar habitat, reaction forces and resulting vibrations do not constitute a significant threat to the habitat structures itself. Hence, considering both the efficacy of plyometric exercise to stimulate the musculoskeletal and cardiovascular systems and the anticipated hypogravity deconditioning in lunar gravity [5], this concept seems to be very promising for long term missions to the Moon, and even more so as it would not require upload of complex and bulky payloads.

Previously published biomechanical and physiological studies in hypogravity have mainly investigated the effects of walking and running and the findings of these studies suggest that lunar gravity will not be sufficient to provide adequate stimuli to prevent deconditioning of the musculoskeletal and cardiovascular systems if no additional countermeasures are implemented [5]. Another hypogravity study conducted in a parabolic flight plane came to the conclusion that during hopping humans learn quickly to adapt to changing gravity conditions by adjusting neuro-motor control of lower leg muscles during hops [21]. In accordance with the present data, Ritzmann et al [21] also showed that peak reaction forces increase with increasing gravity and that contact times decrease with increasing gravity levels. However, in their study Ritzmann et al [21] have not accounted for changes in height of flight–In a parabolic flight plane vertical jumps are restricted through the limited head space (0.7m) and the height of flight was not standardized in their study, making it difficult to draw conclusions on the effects of HoF on jump mechanics in changing hypogravity conditions.

The present data suggest that peak vertical reaction forces are scaled to hopping height and that hopping could therefore be used as a countermeasure to compensate for the reduction of reaction forces during locomotion in hypogravity [24]. Our data suggests that peak vertical reaction forces during submaximal hopping (with a HoF > 15cm; see Fig 2 and supporting information S2 Fig) in lunar gravity can reach the same magnitude as standing in 1G. In Martian gravity conditions submaximal hopping with a HoF of 5cm causes peak reaction forces to reach the same level as walking in 1G, and HoF greater than 15cm generate peak reaction forces that are equal to or greater than peak reaction forces during running at 1G [25]. In addition, our simulation also suggests that submaximal hopping in Martian gravity conditions with HoF > 5cm leads to greater peak vertical reaction forces than walking and running as currently prescribed to ISS astronauts [25]. Therefore, we could expect that if HoF is increased to >30cm, then hopping in lunar gravity conditions would also be superior to walking and running on the ISS, generating high peak vertical GRF (see Fig 2 and supporting information S2 Fig). Submaximal hopping in hypogravity with relatively small HoF is potentially at least equally effective at providing a stimulus to the musculoskeletal system as walking and running at 1G or as currently performed on the ISS. Taking into account that vertical reaction forces can be adjusted through HoF, then increasing HoF to heights that can be achieved during maximal hopping in hypogravity should theoretically be able to generate peak vertical GRF that are even larger as those generated during running and walking in 1G, and thereby provide osteogenic- and muscle-hypertrophic stimuli consistent with mechanostat- and mechano-transduction theories [6, 26, 27].

The relationship between hopping height, changing simulated hypogravity levels and resulting reaction forces (see supporting information S1 Fig) might seem straightforward at first glance. We think, however, that the present experimental study was necessary to show that this seemingly obvious mathematical relationship remains valid in real life with low jump heights and extended legs. For example, and in direct relation to our study, peak vertical GRF are not necessarily always scaled to jump height. Performing maximal countermovement jumps it could be shown that peak vertical GRF are lower than during sub-maximal jumping, as a result of adopting an ankle-only strategy for submaximal, and a hip-knee-ankle strategy in maximal jumps [28]. As a result, correction for knee angle is required for effort (height) to be positively related to peak vertical GRF [29]. Because of this, jump height in fact negatively related to GRF for countermovement jumps. Only when one corrects for effort and knee angle, then the effort is positively related to peak vertical GRF. Thus, it is a necessary step to demonstrate that the expected positive relationship between peak vertical GRF and jump height applies to different (hypo-) gravity environments.

There are several limitations to the present study that need to be addressed. First, the vertical displacement (HoF) of the VTF is limited to 20 cm and thus probably far away from maximal jumps under hypogravity conditions. In addition, the pull-back forces produced by the VTF subject loading system to generate the different levels of G force appear to vary considerably during hop cycles which was unexpected based on the manufacturer claims. This variation is most likely due to viscous properties, although paradoxically these effects were strongest at the nadir and azimuth. Finally, the VTF and its suspension system with its 150cm long strings were manufactured as a tool to study gait and running, in which vertical displacement rarely exceeds 10cm. Whilst lower magnitude jumps were within this, it is possible that larger hops may cause a pendulum-like swing-phase of the body, thereby modifying hop mechanics. Motion capture is required to determine whether this is the case. A future study should also seek to investigate jump heights up to maximal in simulated hypogravity. Supplementary biomechanical measurements would facilitate derivation of joint inverse dynamics, individual motor control strategies and concomitant neuromuscular activation patterns in order to determine whether jumping in excess of 4m in lunar gravity conditions as predicted by Cavagna et al. [22] is possible, and the forces and centre of mass control strategies associated with it are consistent repeated safe landing.

## Conclusions

Our study demonstrates that during plyometric hops in VTF simulated hypogravity, peak reaction forces and flight time are scaled to the height of flight (hopping height/jump height). As a result, they are consistent with the hypothesis that increasing hopping height to provide Earth-like musculoskeletal loading in hypogravity, and thus likely be a potent and low-resource exercise countermeasure on the Lunar surface. However, our data is limited by the relatively low jump heights, inconsistent pull-back forces and the potential for pendulum-like swinging resulting from the suspension system. Therefore, future studies are needed to investigate maximal hopping to determine actual maximal hop heights and the biomechanics associated with them.

## Supporting information

S1 Fig The relationship between gravity, jump height and flight time. (JPG)

S2 Fig Peak vertical GRF. This figure shows individual plots displaying peak vertical GRF for each participant.
(TIFF)

S3 Fig Contact times. This figure shows individual plots displaying contact times for each participant.
(TIFF)

S4 Fig Flight times. This figure shows individual plots displaying flight times for each participant.
(TIFF)

S1 Video Plyometric hopping in simulated hypogravity. This video shows one participant hopping in simulated hypogravity using the vertical treadmill facility. The audio feedback that was used to guide participants during each trial can also be heard.
(MP4)

S1 Table Original data. This table includes original data for all valid hops with the data in the first spread sheet and a codebook in the second one.
(XLSX)
