# Supplementary figures and images for: Hopping in hypogravity—A rationale for a plyometric exercise countermeasure in planetary exploration missions
Source: PLoS One. 2019 Feb 13;14(2):e0211263. doi: 10.1371/journal.pone.0211263 (PMC6373893; doi:10.1371/journal.pone.0211263)

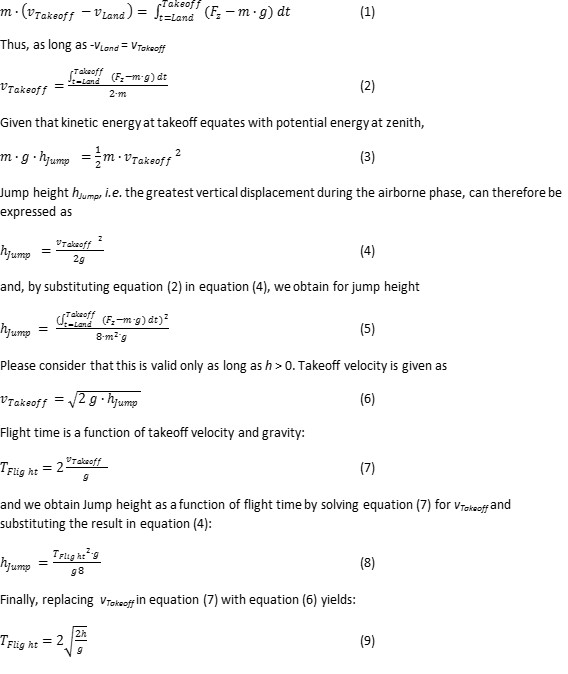

Supplement: S1 Fig — (JPG) [file pone.0211263.s001.jpg]

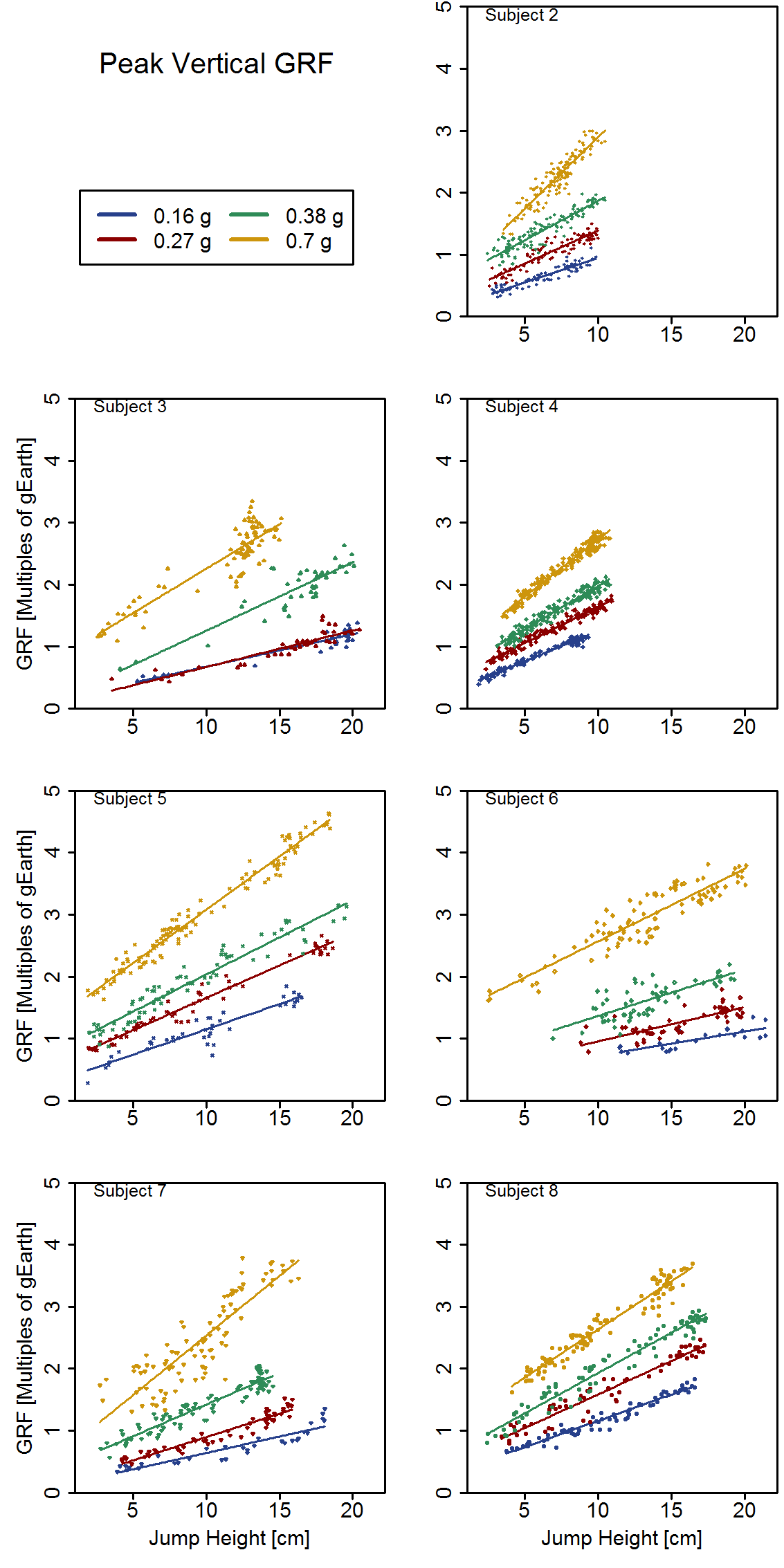

Supplement: S2 Fig — This figure shows individual plots displaying peak vertical GRF for each participant. (TIFF) [file pone.0211263.s002.tiff]

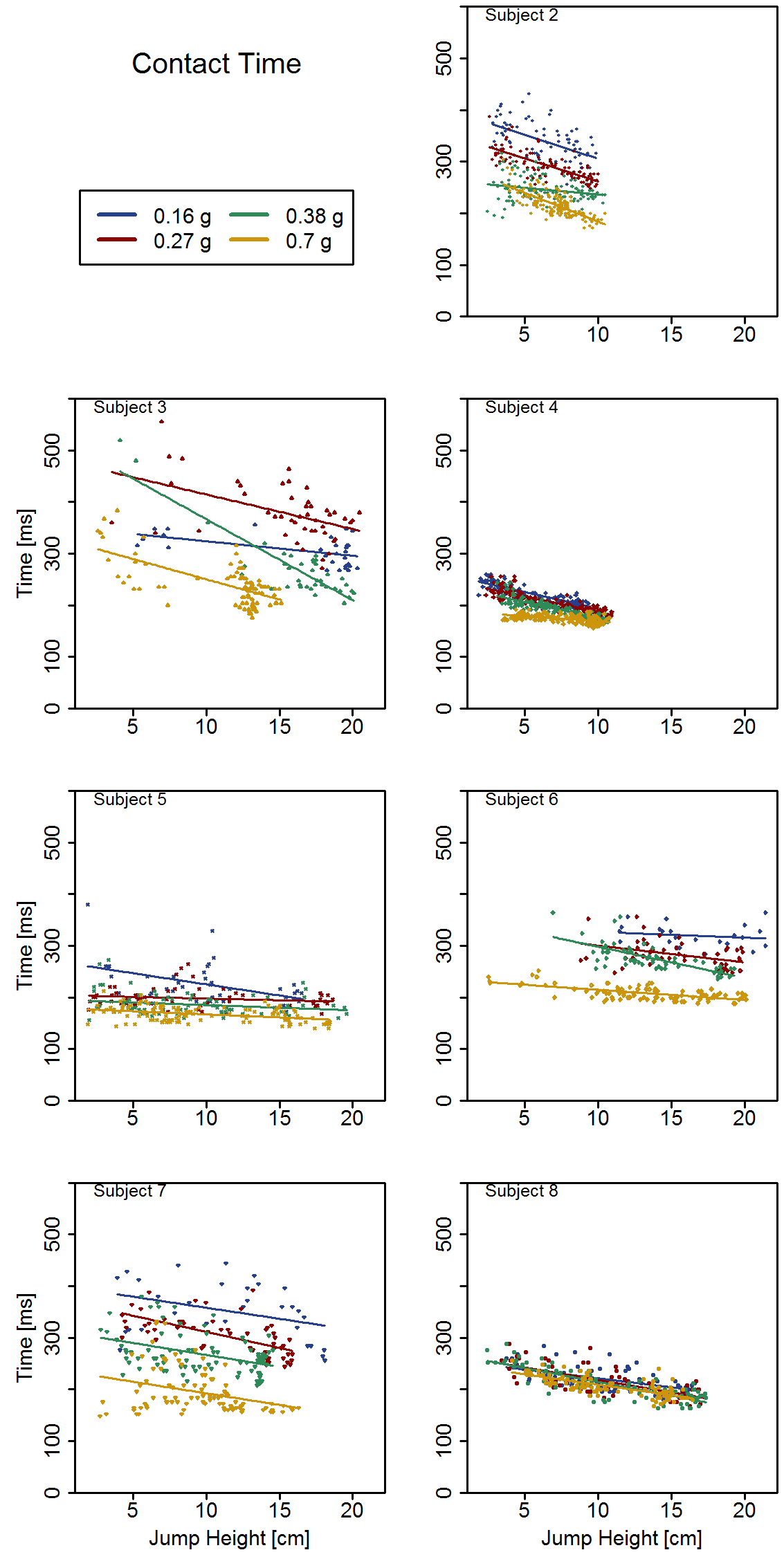

Supplement: S3 Fig — This figure shows individual plots displaying contact times for each participant. (TIFF) [file pone.0211263.s003.tiff]

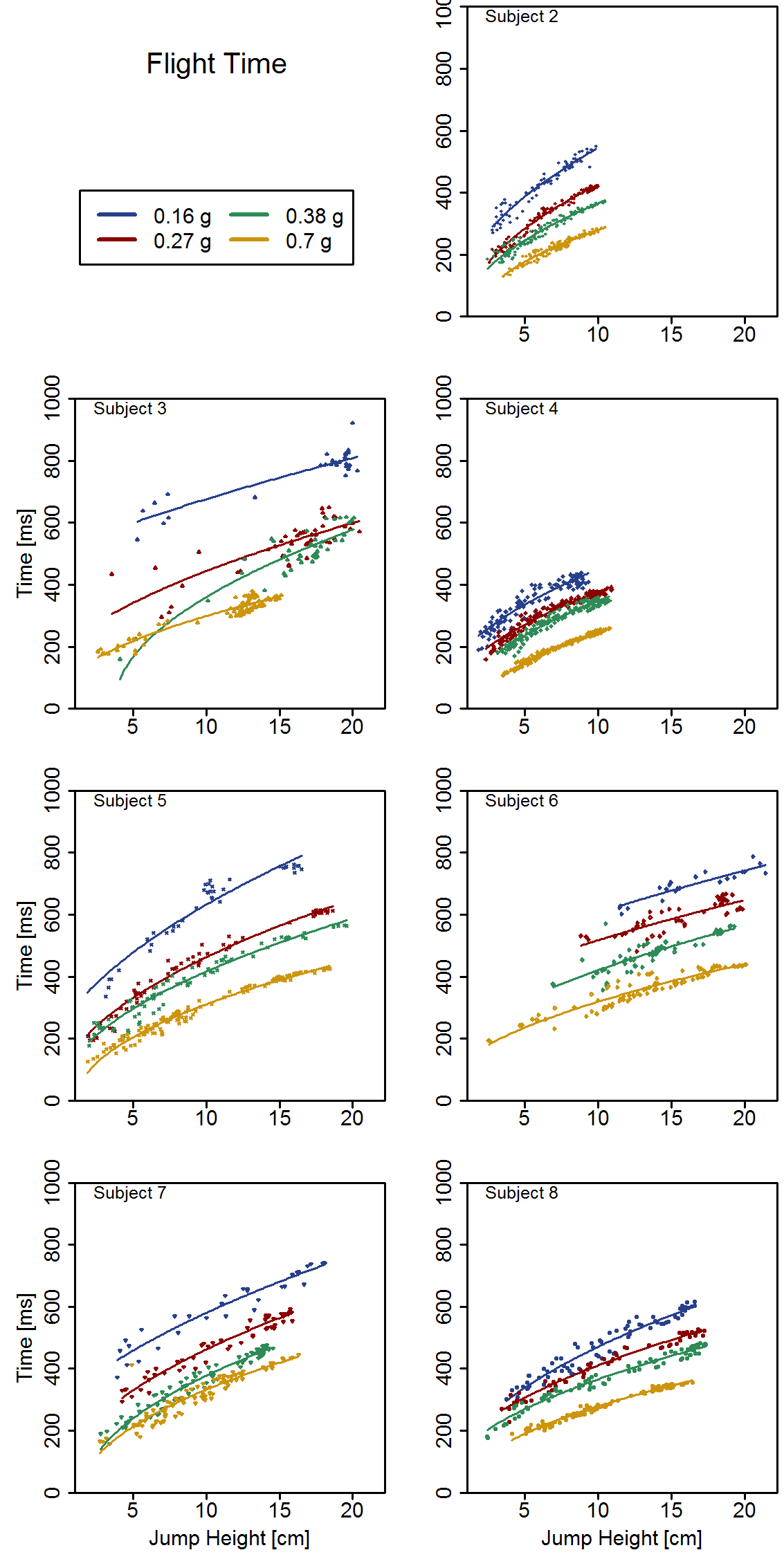

Supplement: S4 Fig — This figure shows individual plots displaying flight times for each participant. (TIFF) [file pone.0211263.s004.tiff]
